# Retraction: MicroRNA-92b inhibits epithelial-mesenchymal transition-induced migration and invasion by targeting Smad3 in nasopharyngeal cancer

**DOI:** 10.18632/oncotarget.28791

**Published:** 2025-12-31

**Authors:** Chong Zhao, Feipeng Zhao, Huajun Feng, Shengen Xu, Gang Qin

**Affiliations:** ^1^Department of Otolaryngology Head and Neck Surgery, The Affiliated Hospital of Southwest Medical University, Luzhou, Sichuan, P.R. China

**This article has been retracted:** Oncotarget has completed its investigation of this paper. Corresponding author, Dr. Gang Qin, requested retraction of the article stating, “some experiments could not reproduce the original results, leading to unreliable conclusions in this paper”. After this request, additional image discrepancies were investigated by Oncotarget. Specifically, internal image duplications were found between Figure 3, panels B and D and Figure 8, panels B and D. In Figure 6B, the GAPDH and Smad3 bands are both partial duplicates of bands found in Figures 4A and 5A of [1]. Figures 7A and 7C contain blots duplicated from Figure 6B of [2]. Figures 8A, 8C, 8E, and 8F contain duplicate blots from Figure 7E of a paper published concurrently [3]. Figure 3B also contains a duplicate image from Figure 8C of [4]. Given these findings, the Editorial decision has been made to retract this article.

Original article: Oncotarget. 2017; 8:91603–91613. 91603-91613. https://doi.org/10.18632/oncotarget.21342
